# The double inhibition of PDK1 and STAT3-Y705 prevents liver metastasis in colorectal cancer

**DOI:** 10.1038/s41598-019-49480-8

**Published:** 2019-09-10

**Authors:** Wenjuan Qin, Yun Tian, Jing Zhang, Wenjian Liu, Qiming Zhou, Sheng Hu, Fei Yang, Li Lu, Haijie Lu, Shuzhong Cui, Lu Wen, Shaozhong Wei

**Affiliations:** 1 Department of Radiation Oncology, Zhongshan Hospital Affiliated by Xiamen University, Xiamen, Fujian China; 2 Department of Abdominal Surgery, The Cancer Hospital Affiliated by Guangzhou Medical University, Guangzhou, Guangdong China; 3 Department of Medical Oncology, Hubei Cancer Hospital, Tongji Medical College, Huazhong University of Science and Technology, Wuhan, Hubei China; 4 Department of Hematological Oncology, Sun Yat-Sen Cancer Center, Guangzhou, Guangdong China; 5 Department of Oncology, The Sixth People’s Hospital, Shenzhen, Guangdong China; 6 Department of Pathology, Hubei Cancer Hospital, Tongji Medical College, Huazhong University of Science and Technology, Wuhan, Hubei China; 7 Department of Gastrointestinal Surgery & Colorectal Cancer Center of Hubei Province, Hubei Cancer Hospital, Tongji Medical College, Huazhong University of Science and Technology, Wuhan, Hubei China; 8 Cancer Center, Union Hospital, Tongji Medical College, Huazhong University of Science and Technology, Wuhan, Hubei China

**Keywords:** Colorectal cancer, Metastasis

## Abstract

As a key glycolysis enzyme, the significance of pyruvate dehydrogenase kinase 1 (PDK1) in the development of colorectal cancer (CRC) remains unknown. This study revealed that the prognosis of CRC patients with high levels of PDK1 was poor, and PDK1 knockdown significantly reduced liver metastasis of CRC in both nude mice and immune competent BALB/C mice. When combined with cryptotanshinone (CPT), an inhibitor of STAT3-p-Y705, the liver metastasis was further inhibited. PDK1 knockdown obviously increased reactive oxygen species level in anoikis conditions and subsequently resulted in an elevated anoikis, but the combination of PDK1 knockdown and CPT showed a reduced effect on anoikis. Based on this discrepancy, the adherence ability of CRC cells to matrix protein fibronectin was further detected. It showed that PDK1 knockdown significantly decreased the adherence of CRC cells to fibronectin when combined with CPT. These results suggest that inhibition of PDK1 can decrease the surviving CRC cells in blood circulation via up-regulation of anoikis, and inhibition of STAT3-p-Y705 can prevent it to settle down on the liver premetastatic niche, which ultimately reduces liver metastasis.

## Introduction

Colorectal cancer (CRC) is the third most common cancer in the world^1^. As the majority of the intestinal mesenteric drainage enters the portal venous system, the liver is the most common site of CRC metastasis^2^. Over 50% of patients with CRC will develop liver metastasis, and more than two-thirds will ultimately die from this metastasis^3^. Hepatic resection of CRC metastasis in patients with isolated metastasis remains the only potential option for cure. Unfortunately, even with modern multi-modal anti-CRC therapy, 70% of patients still develop recurrence in the liver^4^.

Although cancer cells display a diverse range of metabolic profiles, the Warburg effect is a widespread trait^5^. It is noteworthy that mitochondrial function remains intact in most cancers. Pyruvate dehydrogenase kinase 1 (PDK1) is the key glycolysis enzyme that leads to a switch in metabolism from mitochondria-based glucose oxidation to cytoplasm-based glycolysis. A series of studies showed that amplified expression of PDK1 was frequently observed in solid tumors and hematological malignancies, such as ovarian cancer, head and neck cancer, glioma, melanoma, and acute myeloid leukemia^6–10^. Aberrant expression of PDK1 also correlated with unfavorable outcomes in these malignancies. Additionally, PDK1 showed strong cytoplasmic activity in 70% of colorectal tumors, compared with the complete (100%) absence of PDK1 expression in tumor-associated fibroblasts^11^. However, the prognostic value of PDK1 in CRC is still unknown.

Metabolism is intrinsically linked to cell death, as mitochondria plays a critical role in metabolism and apoptosis^12^. Anoikis, as a specific type of apoptosis, is induced by the loss of cell-matrix attachment^13^. Detachment from the matrix stimulates the cells to generate reactive oxygen species (ROS), and excess ROS leads to cell death by stimulating the release of cytochrome c^14^. Thus, antioxidants protect detached cells from anoikis^15^. Moreover, the overexpression of PDKs antagonizes anoikis and prolongs cell survival in suspension in human mammary epithelial cells and melanoma cells^16^. Nevertheless, how PDK1 affects the growth and metastasis of CRC needs to be further clarified.

## Results

### The prognosis of CRC patients with high levels of PDK1 is poor, and knockdown of PDK1 decreases the growth of CRC *in vivo*

**Figure 1:**
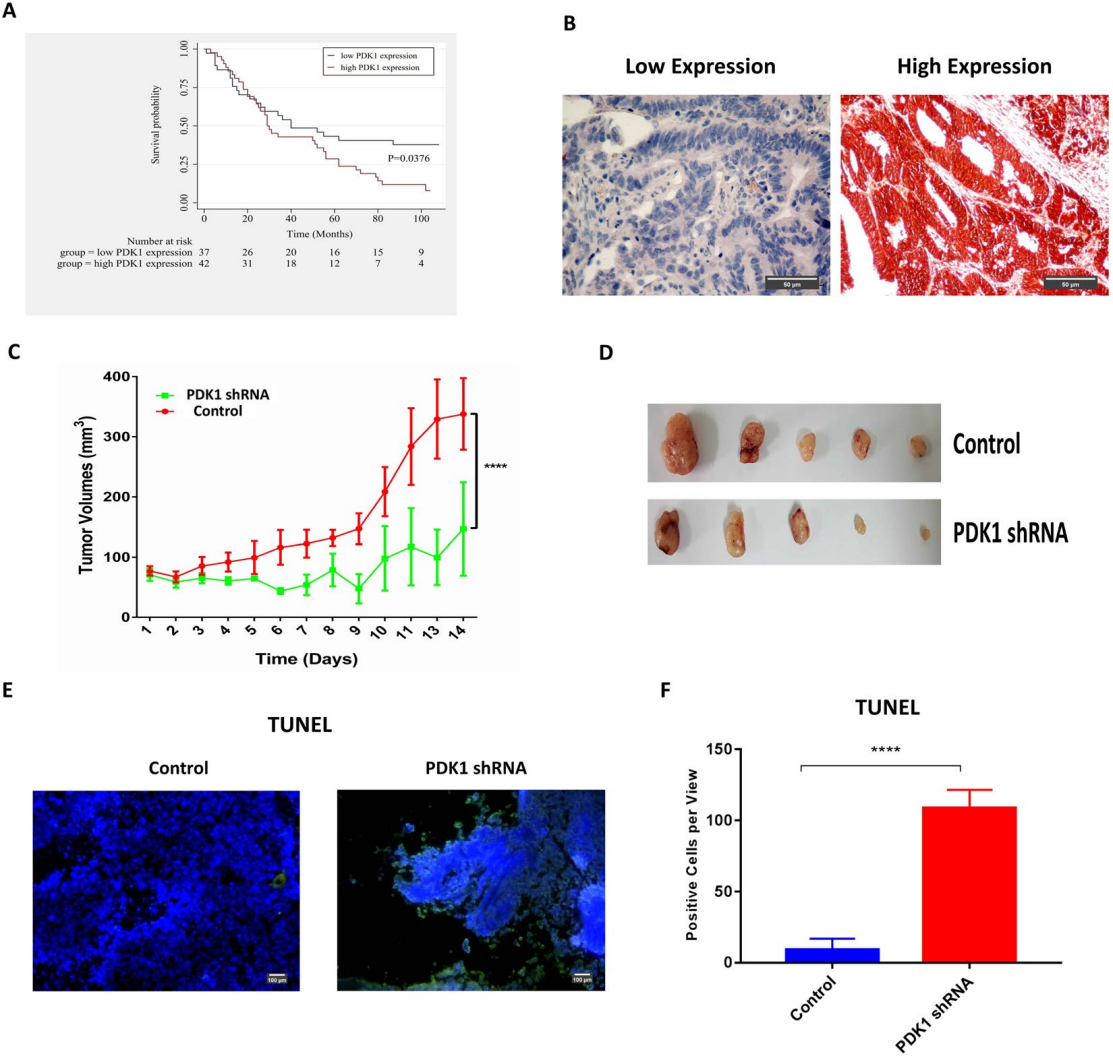
High PDK1 expression is predictive of poor prognosis in CRC patients and promotes tumor growth *in vivo*. (**A**) Overall survival of CRC patients with high PDK1 expression (n = 42) was much shorter than patients with low PDK1 expression (n = 37). (**B**) IHC showed the representative results of low and high PDK1 expression based on the staining index on a tissue microarray. (**C**) Nude mice were subcutaneously injected with 8 × 10^6^ HCT116 cells with or without the transduction of PDK1 shRNA. Silencing PDK1 significantly slowed down the growth of HCT116 xenografts (*P* < 0.0001). (**D**) The gross xenografts were isolated from each nude mouse. (**E**) TUNEL assay showed that silencing PDK1 promoted the apoptosis of CRC cells in residual xenograft of nude mice. (**F**) Histogram displayed the corresponding comparison of the apoptosis cells per view presented in (**E**). Data expressed as mean ± S.D., **** represents *P* < 0.0001.

The primary tumor tissue microarray involved a total of 100 patients with CRC (staged I-IV). Most patients were over 60 years old, and the male-to-female ratio was 1:1.2. As expected, T2 and T3 stage, no lymph node metastasis and left-sided tumors correlated with increased overall survival, compared with T4 stage, lymph node metastasis and right-sided tumors (Fig. S1A–C). Additionally, 37 patients and 42 patients were identified as high PDK1 expression and low PDK1 expression by immunohistochemistry (IHC) respectively, except for unqualified staining on 21 patients. The clinical characteristics for these patients (n = 79) was summarized in Table S1. A Kaplan–Meier survival curve demonstrated that low PDK1 expression was associated with increased overall survival, compared with high PDK1 expression (P = 0.0376, Fig. 1A). Figure 1B showed the representative expression of PDK1 in residual CRC.

Since increased PDK1 expression resulted in a poor prognosis in patients with CRC, we next investigated whether PDK1 contributed to the tumorigenesis in CRC *in vivo*. HCT116 cells transduced with PDK1 shRNA were subcutaneously injected into nude mice. Compared with the control, silencing PDK1 sharply suppressed the growth of HCT116 xenograft (Fig. 1C,D, P < 0.0001). A TUNEL assay was employed to examine the apoptotic cells in xenograft tissue. In line with the result of *in vivo* tumor growth, apoptotic HCT116 cells per field were significantly enhanced by silencing PDK1 (Fig. 1E,F, P < 0.0001). These data suggest that PDK1 plays a crucial role in the growth of CRC.

### Knockdown of PDK1 decreases CRC cell proliferation and STAT3-Y705 phosphorylation

**Figure 2:**
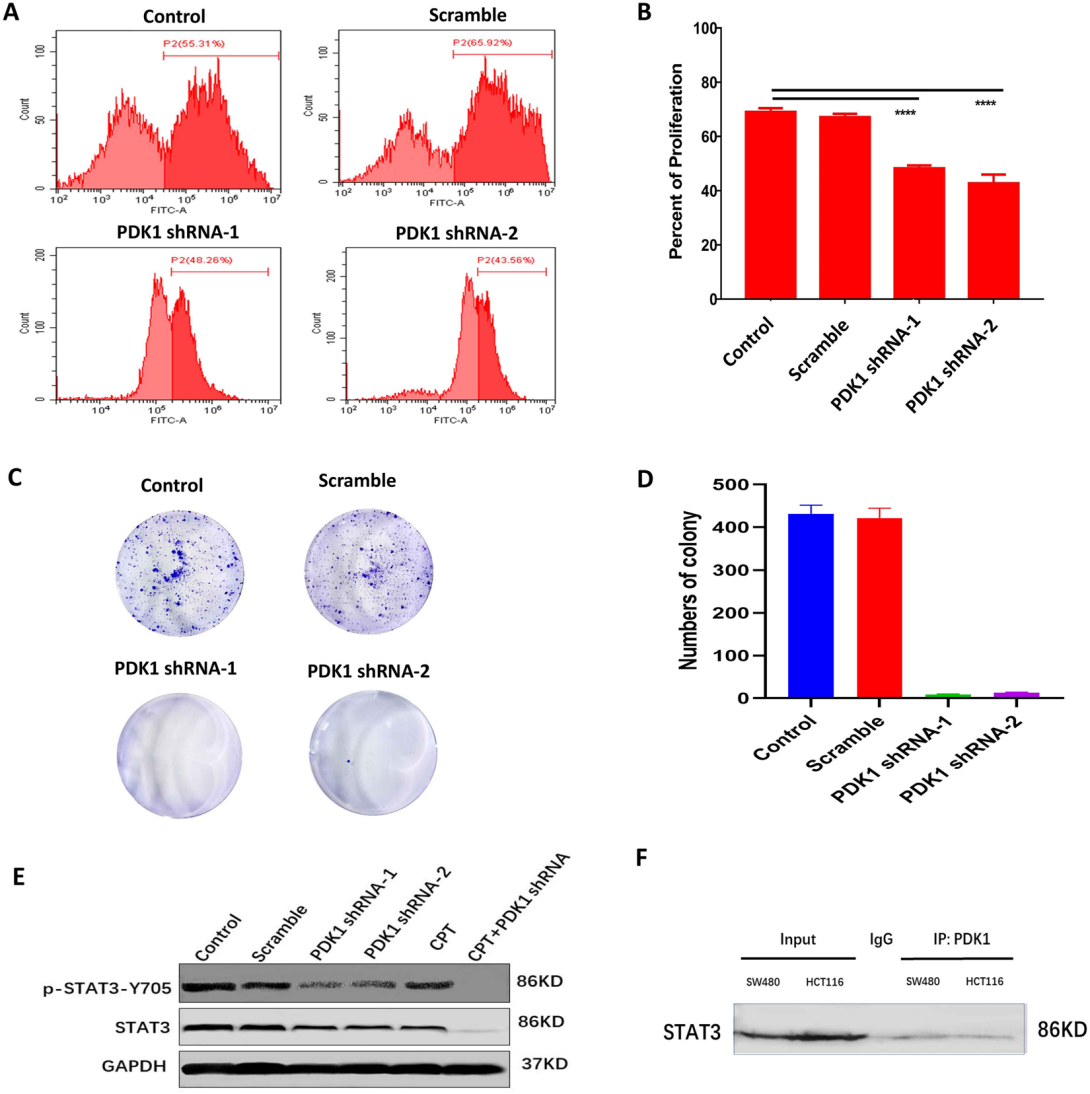
The direct interaction between PDK1 and p-STAT3 may contribute to CRC proliferation. (**A**) EdU incorporation assay showed silencing PDK1 obviously decreased the proliferation of HCT116 cells *in vitro*, compared with the control. (**B**) Histogram displayed the corresponding comparison of the proliferation rate presented in (**A**). (**C**) Colony formation assay demonstrated that knockdown of PDK1 significantly decreased HCT116 cell colony formation *in vitro*. (**D**) Histogram illustrated the corresponding comparison of colony formation numbers presented in (**C**). (**E**) Western blot showed that knockdown of PDK1 significantly reduced STAT3-p-Y705 protein level in HCT116 cells. In particular, the Y705 phosphorylation was completely inhibited by the combination of CPT (a STAT3-p-Y705 inhibitor) and knockdown of PDK1. Three gels were loaded, and blots from different proteins were cropped and grouped into one image with white area separated in between different proteins. The exposure time was 50 s, 30 s, 10 s for p-STAT3, STAT3 and GAPDH, respectively. (**F**) A Co-IP showed PDK1 interacted directly with STAT3 in both HCT116 cells and SW480 cells. Rabbit IgG was served as the control. One gel was loaded, and the blot for STAT3 protein was cropped (exposure time: 40 s). Data expressed as mean ± S.D., **** represents *P* < 0.0001.

Next, we evaluated how PDK1 knockdown affected CRC cell viability and proliferation. CCK-8 assay demonstrated that silencing PDK1 resulted in a significantly higher cytotoxicity in HCT116 and SW480 cells (Fig. S2A). EdU assay was applied to examine whether blockage of PDK1 could inhibit the proliferation of CRC cells. As expected, flow cytometry indicated that blockage of PDK1 by shRNA decreased about 20% proliferation capacity of HCT116 cells, compared with the scramble control (P < 0.0001, Fig. 2A,B). Similarly, silencing PDK1 reduced the proliferation capacity of SW480 cells by about 20% (Fig. S2B). Colony formation assay provided additional evidence for the key role of PDK1 in the tumorigenesis that HCT116 colony numbers were significantly reduced after PDK1 was silenced (Fig. 2C,D, P < 0.0001).

It is generally accepted that STAT3 promotes tumorigenesis by regulating the expression of various target genes, including PDK1^17^. To evaluate the role of endogenous PDK1 in STAT3 signaling, HCT116 cells with or without knockdown of PDK1 were treated with or without p-STAT3-Y705 inhibitor (cryptotanshinone, CPT). Western blots showed that silencing PDK1 obviously suppressed the levels of p-STAT3-Y705, compared with the control and the scrambled construct (Fig. 2E). In particular, the combination of PDK1 shRNA and CPT completely suppressed p-STAT3-Y705 (Figs 2E and S3). To test whether STAT3 interacts directly with PDK1 in CRC, a Co-IP assay was performed using an anti-PDK1 antibody in HCT116 and SW480 cells, which confirmed that a direct PDK1-STAT3 interaction indeed existed in these two CRC cells (Fig. 2F).

### Silencing PDK1 and inhibiting p-STAT3-Y705 significantly reduces liver metastasis of CRC in both immune deficient and immune competent mice

**Figure 3:**
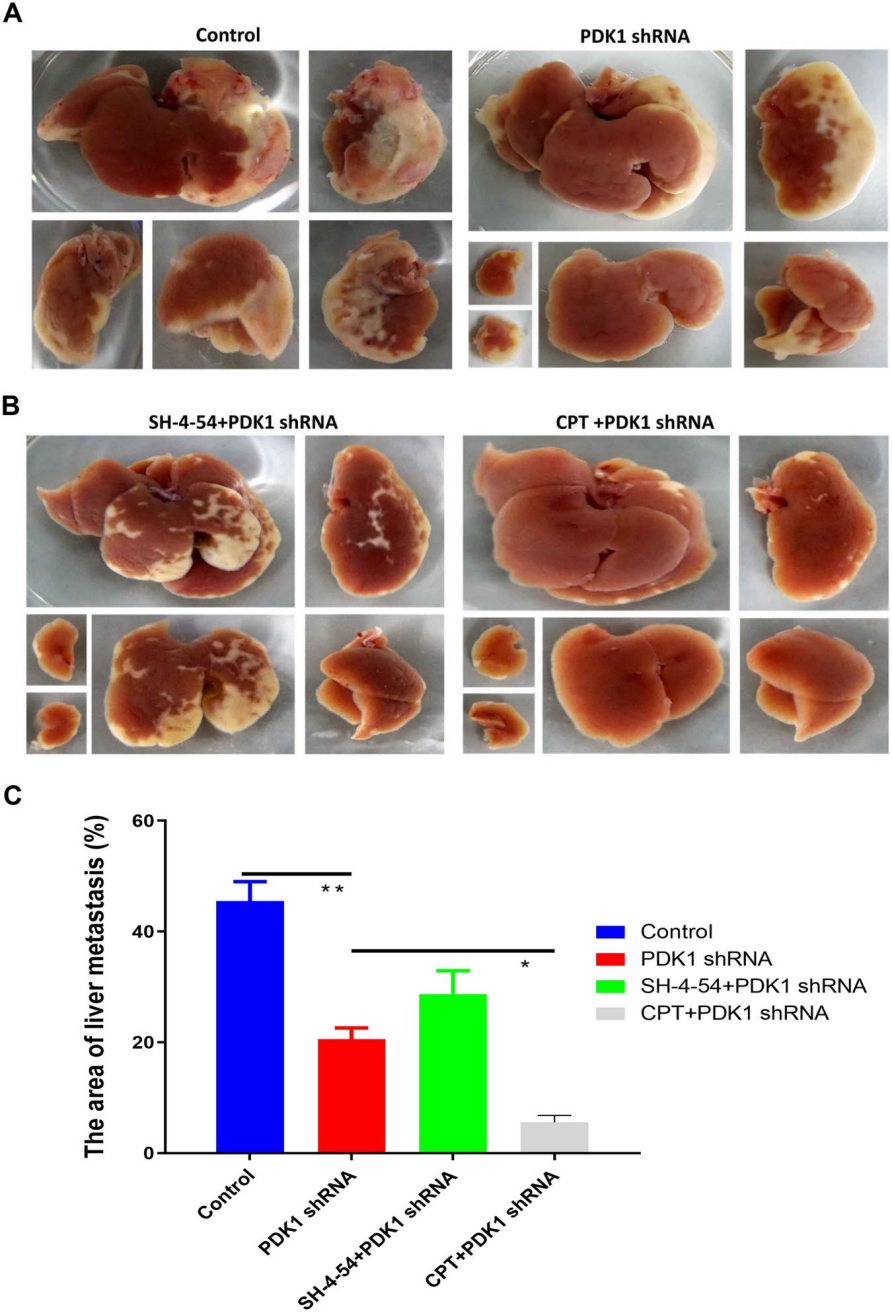
Knockdown of PDK1 and inhibiting p-STAT3-Y705 decreased liver metastasis of colon cancer in the nude mice model. (**A**) 5 × 10^6^ HCT116 cells with or without PDK1 shRNA transduction were injected into the spleens of nude mice under isoflurane anesthesia on day 1, respectively. On day 9, the nude mice were sacrificed, and the liver metastatic area was measured by ImageJ software. Compared with the control group, knockdown of PDK1 significantly inhibited liver metastasis of HCT116 cells. (**B**) SH-4-54 (an inhibitor of total STAT3, 0.2 mg/d/mouse) or CPT (0.2 mg/d/mouse) were injected (i.p.) every other day (day 1, 3, 5, 7) for a total of four times. The results showed that CPT plus knockdown of PDK1 markedly decreased the liver metastasis area, compared with the combination of SH-4-54 and knockdown of PDK1. (**C**) Histogram indicated the corresponding comparison of liver metastasis areas presented in (**A**,**B**). Data expressed as mean ± S.D., * represents *P* < 0.05, ** represents *P* < 0.01.

**Figure 4:**
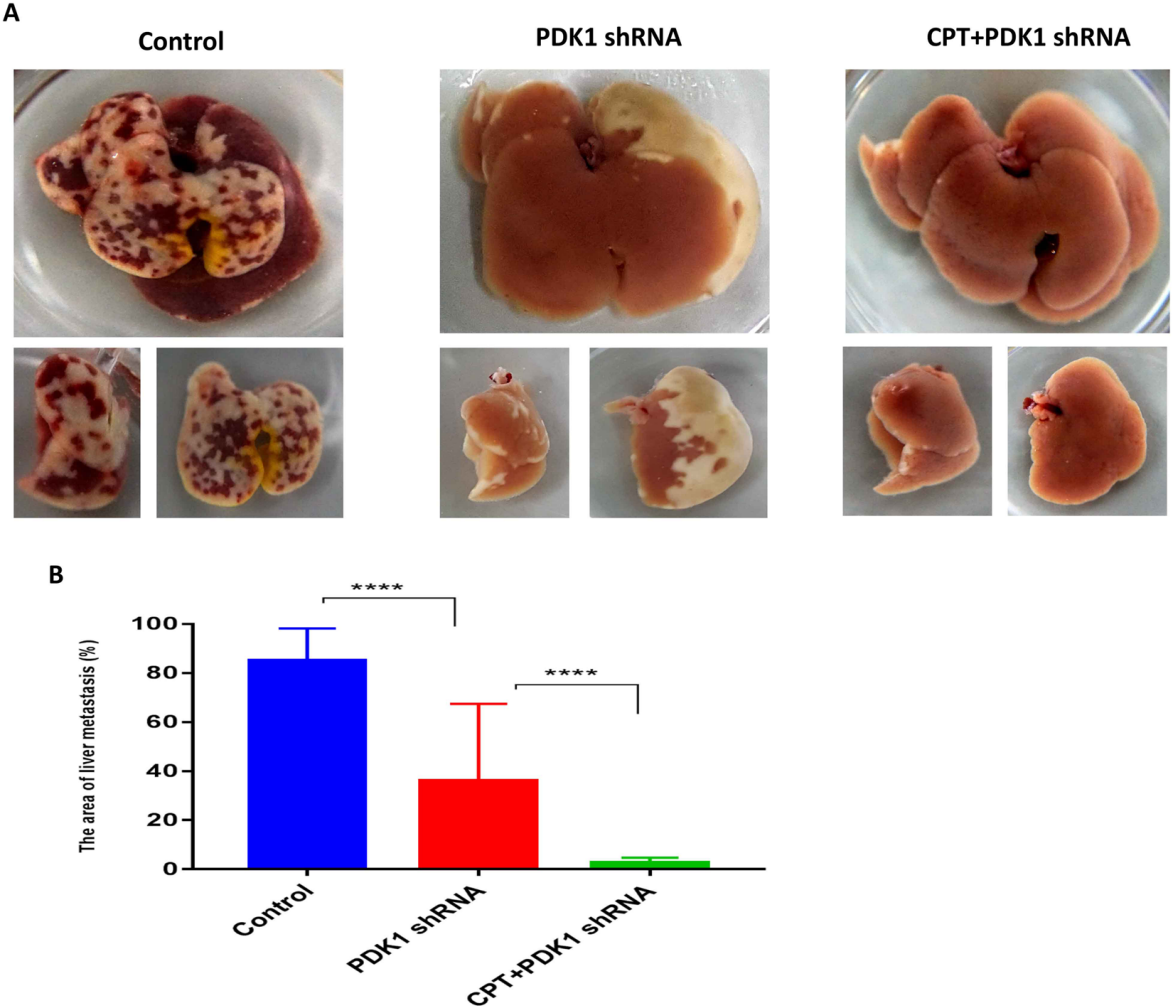
The combination of CPT and silencing PDK1 significantly inhibited liver metastasis of CRC by down-regulating p-STAT3-Y705 in the immune competent mice model. (**A**) 5 × 10^6^ HCT116 cells with or without PDK1 shRNA transduction were injected into the spleens of BALB/C mice under isoflurane anesthesia, respectively. CPT (0.2 mg/d/mouse) were injected (i.p.) every other day (day 1, 3, 5, 7) for a total of four times. On day 9, the mice were sacrificed, and the liver metastatic area was measured by ImageJ software. The combination of CPT and PDK1 shRNA treatment led to a smallest liver metastasis area, compared with the control or PDK1 shRNA treatment alone. (**B**) Histogram indicated the corresponding comparison of liver metastasis areas presented in (**A**). Data expressed as mean ± S.D., **** represents *P* < 0.0001.

It has remained unclear whether PDK1 is involved in the liver metastasis of CRC. For this purpose, we established a liver metastasis model in nude mice by performing intrasplenic injection of HCT116 cells without or with silencing PDK1. The results showed that the liver metastasis area was significantly reduced when PDK1 was silenced, compared with the non-transduced control (Fig. 3A, P < 0.01). Meanwhile, we further investigated whether inhibiting STAT3 phosphorylation could influence the efficacy of silencing PDK1 treatment. The results showed that the combined strategy of silencing PDK1 and CPT resulted in a sharply smaller area of liver metastasis, compared with silencing PDK1 alone. However, the combination of silencing PDK1 and total STAT3 inhibitor (SH-4-54) unexpectedly led to a worse efficacy than silencing PDK1 alone (Fig. 3B,C). To validate its efficacy in immune competent hosts, BALB/C mice were used to establish the liver metastasis model by the above same method. It showed a similar result with the immune deficient mice model (Fig. 4A,B), confirming that silencing PDK1 significantly inhibited liver metastasis in CRC, and the simultaneous administration of CPT further improved its efficacy by down-regulation of p-STAT3-Y705 (P < 0.01), which was independent of host immune status. To strengthen the clinical translation, we further examined the efficacy of CPT with or without an unspecific PDK1 inhibitor (dichloroacetate, DCA), which also demonstrated that CPT or CPT plus DCA significantly reduced the liver metastasis of colon cancer, compared with the control group, moreover, the combined efficacy of CPT and DCA was better than that of single CPT treatment (Fig. S4).

### The combinative effect of PDK1 knockdown and CPT on inhibiting liver metastasis was partly mediated by the sensitization to anoikis

**Figure 5:**
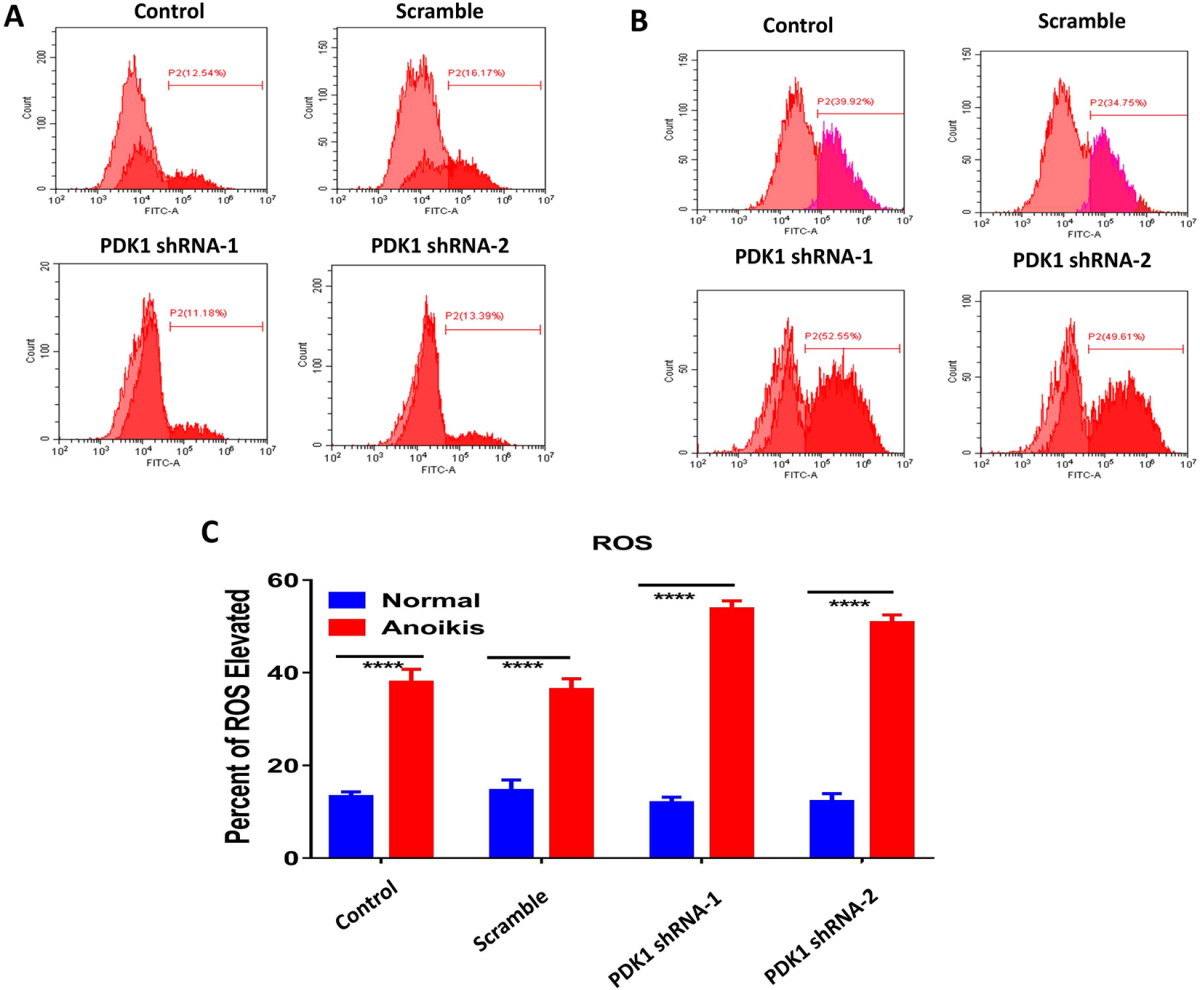
Silencing PDK1 significantly elevates the cytoplasmic ROS level. (**A**) HCT116 cells with or without the transduction of a PDK1 shRNA were cultured in normal condition. Flow cytometry showed ROS levels had no obvious difference regardless of PDK1 status. (**B**) As indicated in (**A**), cells were cultured in matrix detachment. ROS levels were significantly enhanced by silencing PDK1, compared with the controls. (**C**) Histogram indicated the corresponding comparison of ROS levels presented in normal and anoikis culture conditions in (**A**,**B**). Data expressed as mean ± S.D., **** represents *P* < 0.0001.

**Figure 6:**
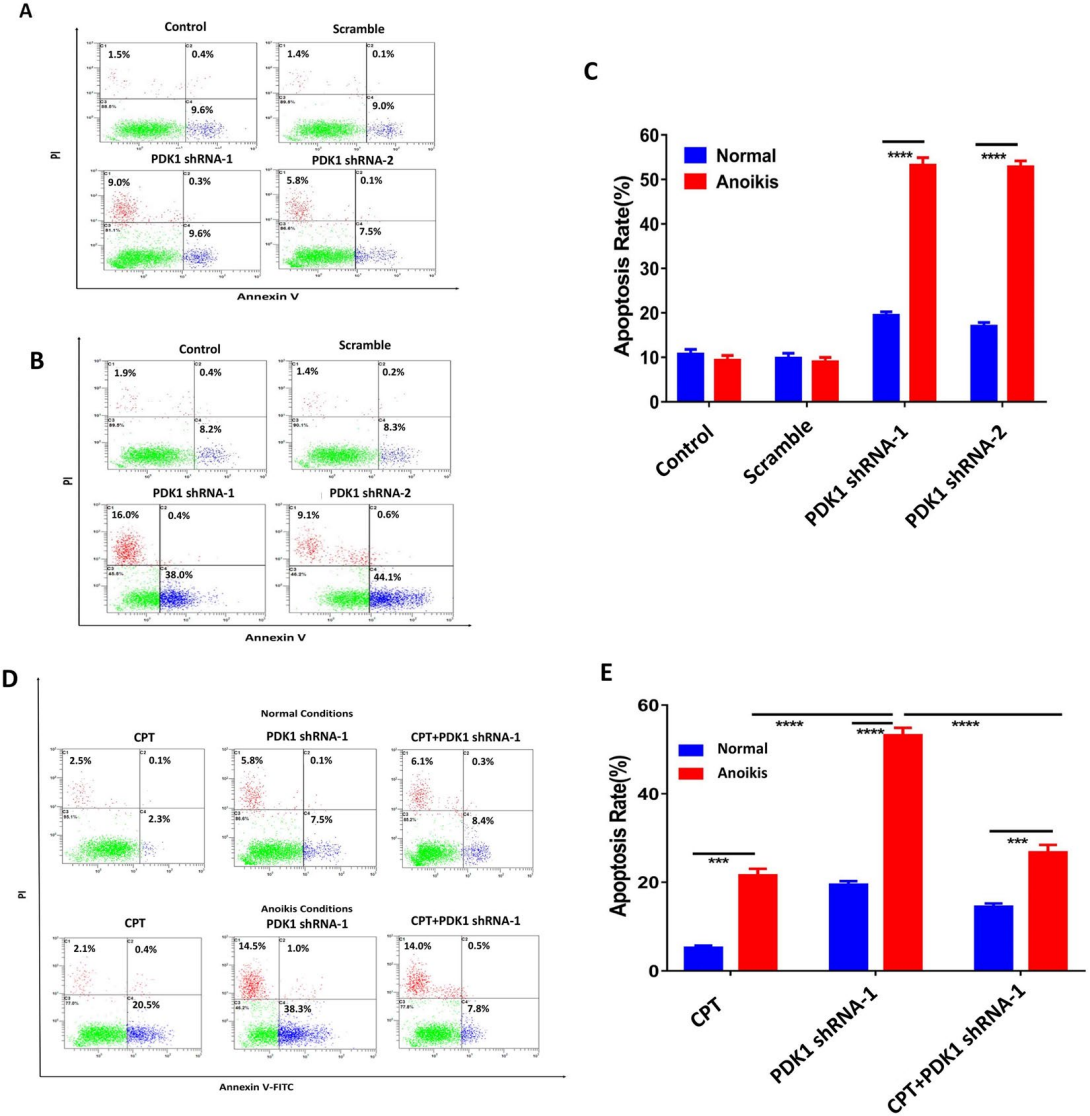
Anoikis partly contributes to the *in vivo* effect of the combination of knockdown of PDK1 and CPT. (**A**) HCT116 cells with or without the transduction of a PDK1 shRNA were seeded into 6-well plates and cultured in normal conditions. Then the cells were stained with AnnexinV-FITC/PI. Flow cytometry showed that the apoptosis rate was much higher after PDK1 was silenced. (**B**) As indicated in (**A**), the cells were seeded into 6-well plates coated with poly-HEMA. Flow cytometry showed that the apoptotic rate of HCT116 cells stably transduced with PDK1 was significantly higher than the other two controls. (**C**) Histogram indicated the corresponding comparison of the apoptosis rate presented in (**A**,**B**). (**D**) The results showed knockdown of PDK1 strongly sensitized CRC to anoikis; however, the anoikis rate was significantly reduced when PDK1 silencing was combined with CPT. (**E**) Histogram indicated the corresponding comparison of the apoptosis results presented in both normal condition and suspending condition in (**D**). Data expressed as mean ± S.D., *** represents *P* < 0.001, and **** represents *P* < 0.0001.

Anoikis resistance is one of the major factors that contribute to cancer metastasis^18^. Some studies indicated that ROS regulated anoikis resistance either positively or negatively, depending on cell context^19,20^. To find out the potential reason why knockdown of PDK1 suppressed liver metastasis in CRC, ROS levels of HCT116 cells were compared under different culture conditions. Firstly, HCT116 cells and HCT116 cells stably transduced with a negative vector, PDK1 shRNA-1 or shRNA-2, were seeded into 6-well plates. After 3 days of *in vitro* normal culture, the cellular ROS level was measured by flow cytometry. As shown in Fig. 5A, the ROS level did not show an obvious difference between the tested and control cells (both were about 15%, P > 0.05). Secondly, a poly-HEMA culture was used to establish an anoikis model to measure ROS according to the above same method, which displayed that the ROS level of HCT116 cells with knockdown of PDK1 rose sharply to approximately 50%, which was significantly higher than that in controls (about 35%) (Fig. 5B,C). That is, ROS level of HCT116 cells was elevated when they were cultured in anoikis status, in particular, knockdown of PDK1 significantly correlated with a higher ROS level in anoikis status. Next, we examined the apoptotic rate of HCT116 cells in these two culture conditions. For normal culture, the apoptotic rate was about 10% at baseline, and it rose to approximately 18% after PDK1 was silenced (P < 0.01, Fig. 6A). As for anoikis culture, the baseline apoptotic rate was similar with that in normal culture, however, the value was about 5 times higher when PDK1 was silenced (P < 0.0001, Fig. 6B,C). Moreover, silencing PDK1 significantly inhibited the expression of PDK1 and upregulated the expression of cleaved caspase-3 by western blot (Fig. S5A). We further examined the expression of antioxidative genes (SOD3, PRDX1) in HCT 116 cells with or without PDK1 knockdown in these two culture conditions. In normal culture, the expression of SOD3 and PRDX1 was downregulated when the PDK1 was silenced (Fig. S5B), but knockdown of PDK1 upregulated the expression of these two genes in anoikis condition (Fig. S5C), possibly resulting from a protective reaction against anoikis. These results indicated that knockdown of PDK1 promoted the apoptosis of HCT116 cells in anoikis condition by upregulation of cleaved caspase-3. Though PDK1 knockdown or CPT treatment upregulated the apoptosis of HCT116 cells in anoikis condition, the combination of knockdown of PDK1 plus CPT significantly reduced the apoptotic rate of HCT116 cells, compared with knockdown of PDK1 alone (Fig. 6D,E). Thus, additional mechanisms may be involved in the regulation of liver metastasis.

### Knockdown of PDK1 plus CPT significantly reduces liver metastasis of CRC by downregulating the adherence capacity via inhibiting STAT3-p-Y705

**Figure 7:**
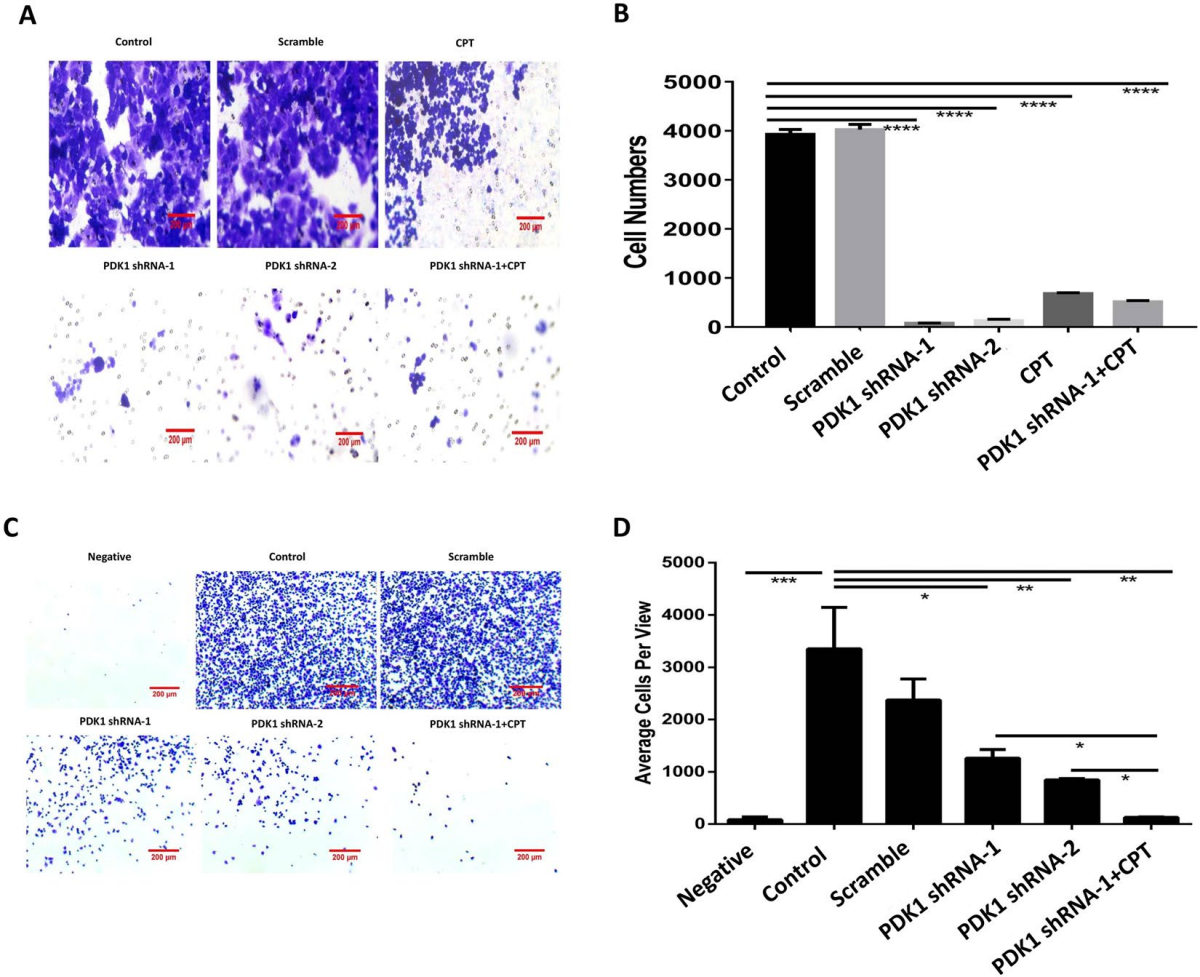
Knockdown of PDK1 plus CPT significantly reduces liver metastasis in CRC by downregulating the adherence capacity via inhibiting STAT3-p-Y705. (**A**) Transwell assay showed that the knockdown of PDK1 or the combination of CPT and silencing PDK1 significantly decreased migration capacity of liver metastatic HCT116 cells. (**B**) Histogram indicated the corresponding comparison of migration capacity presented in (**A**). (**C**) The liver metastatic HCT116 cells were seeded into a 6-well plate coated with fibronectin. The adherence assay showed that the knockdown of PDK1 significantly decreased the chemotaxis of liver metastatic HCT116 cells. In particular, the combination of CPT and knockdown of PDK1 resulted in a lowest cell adherence capacity. (**D**) Histogram indicated the corresponding comparison of the adherent cells presented in (**C**). Data expressed as mean ± S.D., * represents *P* < 0.05, ** represents *P* < 0.01, *** represents *P* < 0.001, and **** represents *P* < 0.0001.

Cancer cell invasion and adherence are the two leading causes for metastasis^21^, so we next further investigated the effect of inhibiting STAT3-p-Y705 on cell invasion and adherence. Transwell experiment showed that the migration cells were less than 800 for silencing PDK1 and/or CPT treatment, but the numbers were about 4000 for the two controls, suggesting that inhibiting PDK1 and/or STAT3-Y705 phosphorylation significantly decreased invasion capacity of liver metastatic HCT116 cells (Fig. 7A,B, P < 0.0001). In addition, the average adherent cell numbers per view were less than 1500 for silencing PDK1 or the combination of CPT and silencing PDK1, however, the numbers were more than 3000 for the control without any treatment. In particular, the combination of these two treatments resulted in a significantly lower cell adherence capacity, compared with silencing PDK1 alone (P < 0.05, Fig. 7C,D), which means that the combined treatment significantly sensitizes liver metastatic HCT116 cells to a reduced adherence capacity and subsequently suppresses liver metastasis.

## Discussion

Aerobic glycolysis in cancer cells is needed to supply glycolytic intermediates for anabolic support for cell proliferation, but it remains unclear why pyruvate is preferentially disposed of as secreted lactate rather than utilized by the tricarboxylic acid (TCA) cycle. Cancer cells often increase their consumption of glutamine to replenish the TCA cycle^22^. Therefore, it appears that tumors aim to restrict the entry of pyruvate into the mitochondrial oxidative metabolism. Silencing PDK4 induced chemotherapy-associated damage in hepatocytes and colon cancer^23^, and decreased the cellular migration and invasion in colon cancer cells^24^, whereas up-regulation of PDK4 increased the resistance of hepatocytes and colon cancer against chemotherapy induced toxicity^23^. These results suggest that PDK4 is involved in the development and drug resistance in CRC, though high hepatic, but not tumoural expression of PDK4 is associated with improved survival in patients undergoing surgical operation of resectable colorectal liver metastases^23^. In addition, the knockdown of PDKs in cancer cells stimulates glucose oxidation and ROS production, thus restoring the susceptibility of the cells to anoikis and reducing their ability to metastasize in normal cell line and melanoma^16^, which suggests that cancer cells may purposefully select aerobic glycolysis to reduce ROS production, avoid ROS mediated anoikis, and ultimately promote metastasis. Consistent with the study of Kamarajugadda *et al*.^16^, the present study showed that the knockdown of PDK1 sensitized CRC to anoikis by increasing ROS production.

Since acquiring anoikis resistance is the first step toward metastasis^25^, we next examined the significance of PDK1 in CRC metastasis. It demonstrated that silencing PDK1 in CRC cells significantly reduced liver metastasis in both nude mice and immune competent mice. In particular, the addition of CPT further improved its efficacy. However, the combined treatment led to a lower anoikis than knockdown of PDK1 alone. This discrepancy indicated that an additional mechanism contributed to the synergistic effect. Extracellular matrix (ECM), such as fibronectin, collagen, and laminins, was a major component of tumor microenvironment that played an essential role in tumor metastasis^26–28^. In addition, changes in the production and organization of fibronectin in the ECM contributed to a favorable pre-metastatic niche^29^, which dictated the pattern of metastatic spread. Furthermore, Barbazán *et al*. reported that luminal side of liver blood vessels contained fibronectin deposits that were enriched in mice bearing CRC and human livers affected with metastases, more importantly, CRC cells attached to endothelial fibronectin deposits via talin1, a major component of focal adhesions, resulting in liver metastasis formation *in vivo*^30^. In this study, we further performed cell invasion and adherence assays, and found that CPT significantly decreased migration and adherence capacity of liver metastatic CRC cells by inhibiting STAT3-p-Y705, which is critical to prevent tumors from colonization in liver. This gives a reasonable explanation for the discrepancy that the combined treatment displays a lower anoikis, but results in a better efficacy. Thus, these results suggest that it may be a promising strategy to combine PDK1 and STAT3-p-Y705 inhibitor for effectively reducing CRC metastasis.

The expression of PDK1 was considered to correlate with a variety of tumor progression markers and with patient prognosis. The inhibition of PDK1 with DCA has been investigated in several clinical trials, including trials for glioma and refractory metastatic breast cancer (ClinicalTrials.gov Identifier: NCT01111097, NCT01386632, and NCT01029925)^31^. However, the high effective dosage and side effects have limited the application of DCA^32^. To date, no clinical trial related to PDK1 inhibitors has been carried out for CRC. Therefore, it is necessary to explore new potent PDK1 inhibitors with a better safety profile and fewer side effects and to examine the activity of these inhibitors in CRC. Nevertheless, a major problem that must be taken into consideration for future application is that the inhibition of PDK1 can be only useful up to a certain point and may not be sufficient to cause extensive apoptosis in cancer cells alone^33^.

STAT3 is a key transcription factor for oncogenesis by upregulating several genes that control cell survival, angiogenesis, apoptosis, migration and cell cycle^34^. Demaria *et al*. demonstrated a non-canonical role of STAT3 signaling involving the Warburg effect in a metabolomics analysis, in which the induction of aerobic glycolysis was an important component of STAT3 pro-oncogenic activities^35^. Moreover, STAT3 inactivation reversed the glycolytic shift by down-regulating key enzymes (including PDK) and then inhibited tumor growth^36^. Our findings showed a direct interaction between PDK1 and STAT3 that silencing PDK1 significantly down-regulated p-STAT3-Y705 in CRC cells, suggesting a significant role of STAT3 signaling on cancer metabolism.

**Figure 8:**
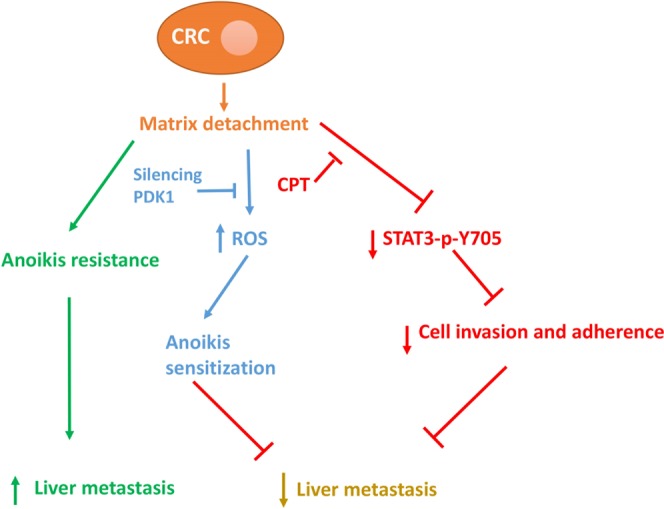
The potential mechanism of knockdown of PDK1 and CPT on inhibiting liver metastasis in CRC.

Collectively, we suggest a model (Fig. 8) for the mechanism of inhibiting liver metastasis mediated by the combined treatment of CPT and knockdown of PDK1. Cancer cells will undergo anoikis when they break away from where they first form, and silencing PDK1 can reduce the numbers of cancer cells in the lymph system or bloodstream by elevated anoikis resulting from upregulation of ROS. Though some cancer cells inevitably spread to the liver, simultaneous inhibition of STAT3-p-Y705 significantly decreases the adherence of cell-matrix, and ultimately reduces the liver metastasis in CRC.

## Materials and Methods

### Cell culture and tissue microarray

The HCT116 and SW480 CRC cell lines were obtained from ATCC and maintained in high-glucose DMEM supplemented with 10% fetal calf serum (*FCS*, *HyColony*, *Logan*, *UT*). A tissue microarray involving the clinicopathological information of 100 patients with CRC cancer was purchased from Shanghai Outdo Biotech Company & National Engineering Center for Biochip Design and Engineering, China.

### IHC staining

IHC was performed as described previously^37^. Briefly, tissue sections underwent deparaffinization in xylene, followed by rehydration through graded ethanol at room temperature, which then were heated in a 1 mmol/L ethylenediaminetetraacetic acid (EDTA) buffer (water bath, 96–98 °C) for 15 min for antigen retrieval. Peroxidase activity was quenched with 0.3% hydrogen peroxide. The primary anti-PDK1 antibody (*cat#: 10026-1-AP*, *Proteintech Group*, *Inc*, *Rosemont*, *IL*, *USA*) was used at a dilution of 1:100. Next, the slide was incubated with a biotin-labeled secondary antibody for 30 min and streptavidin-horseradish peroxidase for 30 min. Diaminobenzidine was used as the chromagen and slide was counterstained with hematoxylin. A staining index (values, 0–2) that was determined by multiplying the score for staining intensity with the score for positive area was employed to determine the PDK1 expression level in CRC tissues. Scores ≥9 indicated high expression, and scores <9 indicated low expression.

### TUNEL assay

After deparaffinization with xylene and rehydration through graded alcohols, proteinase K were added to the sections for 30 min at room temperature. After washing 3 times, the TUNEL detection solution was prepared according to the manufacturer’s instructions (*Beyotime*, *China*). The solution was added to the sections for 60 min of incubation at 37 °C. Apoptosis was examined under a fluorescence microscope (*Olympus*, *Japan*).

### Colony formation assay

Five hundred cells were seeded in each well of a 6-well plate and maintained in 2 ml of DMEM. The medium was changed every 3 days. Colonies were fixed with 4% poly-paraformaldehyde and stained with 0.25% crystal violet on day 14, and the numbers of colonies were manually counted.

### CCK-8 assay

Cell cytotoxicity was determined using CCK-8 kits. Cells were seeded in 96-well plates at a density of 2 × 10^3^ cells. After 3-day culture, 10 μl CCK-8 reagent was added to each well and incubated at 37 °C for another 1 h. Optical density values were measured at 450 nm using the microplate reader.

### Cell invasion

Cell invasion was determined using a Transwell cell culture chamber (*Becton-Dickinson; NJ*, *USA*). A total of 1 × 10^4^ cells cultured in serum-free DMEM containing 0.1% BSA were added to the upper chamber and incubated for 24 h in the presence of 20% FCS in the lower chamber. The cells that migrated to the reverse side of a filter with an 8 μm pore were fixed with 4% poly-paraformaldehyde and stained with crystal violet after 24 h. The data represented an average of three independent experiments that were conducted in duplicate.

### Cell proliferation, ROS level and apoptosis assay

For EdU incorporation assay, the procedure was carried out according to the manufacturer’s instructions with EdU detection kits (*NanJing KeyGen Biotech Co*., *Ltd*.). Briefly, HCT116 cells or SW480 cells (with or without transfection of PDK1 shRNA) were seeded in 6-well plates. After 24 h, 2 × EdU working solution (20 µM) was added to the 6-well plates for 2 h culture at 37 °C, then the medium was discarded, followed by the addition of 1 ml fixing solution for 15 min at room temperature. Next, the fixing solution was discarded, and cells were washed 3 times, followed by the addition of 1 ml 0.3% Triton X-100 for 15 min. Finally, cells were cultured with 500 µl Click Reaction Buffer for 30 min at dark.

For ROS assay, HCT116 cells with or without transfection of PDK1 shRNA were cultured at both normal condition and anoikis condition for 24 h in 6-well plates. In the anoikis model, 6-well plates were coated with poly-HEMA (*Sigma-Aldrich*, *USA*). Then, the complete medium was discarded, followed by the addition of 1 ml serum free medium containing 2′,7′-dichlorodihydrofluorescein diacetate (final concentration: 10 mmol/L). After 20 min culture, cells were washed with serum free medium for 3 times. Next, cells were digested with trypsin, and centrifuged at 500 g, 5 min. The supernatant was discarded, followed by the addition of 200 µl serum free medium.

For apoptosis assay, HCT116 cells were cultured with or without CPT treatment (10 μM) at normal condition or anoikis condition for 24 h. Then, cells were digested by trypsin, and centrifuged at 500 g, 5 min, followed by re-suspension with 195 μl Annexin V-FITC binding buffer, and 5 μl Annexin V-FITC, 10 µl PI at dark for 20 min.

Cells in these assays were finally subjected to flow cytometry (*Cytomics FC500 Flow Cytometer*, *260 Beckman Coulter*) and finally analyzed by FlowJoTM version 10 (*Tree Star*, *Inc*., *Ashland*, *OR*, *USA*).

### Cell adhesion

First, 24-well plates were coated with human fibronectin (*Yeasen*, *China*). CRC cells that were stably transduced with a PDK1 shRNA were incubated with or without 10 µM CPT and were then washed and counted. Equal numbers of cells were seeded into each well of the fibronectin-coated 24-well plate. After 30 min of incubation, the plate was washed, and the supernatant was discarded. The cells were fixed with 4% poly-paraformaldehyde and stained with crystal violet. The images were captured under a microscope and analyzed by ImageJ software.

### Western blotting analyses

Cellular lysates were prepared by suspending 1 × 10^6^ cells in 100 µl of RIPA lysis buffer (1 × PBS, 1% NP-40, 0.5% sodium deoxycholate, and 0.1% SDS) that was supplemented with 10 mM β-glycerophosphate, 1 mM sodium orthovanadate, 10 mM NaF, and 1 mM phenylmethylsulfonyl fluoride. The cells were extracted on ice for 30 min. The proteins were electro-transferred to nitrocellulose membranes (*Millipore Corp*., *Bedford*, *MA*), and specific proteins were detected using chemiluminescence. Anti-PDK1 antibody (*cat#: 10026-1-AP*), anti-Akt antibody (*cat#: 10176-2-AP*), anti-p-Akt (S473) antibody (*cat#: 66444-1-Ig*), anti-SOD3 antibody (*cat#: 14316-1-AP*), anti- GAPDH antibody (*cat#: 60004-1-Ig*), anti-PRDX1 antibody (*cat#: 15816-1-AP*) were purchased from Proteintech Group, Inc, Rosemont, IL, USA. Anti-STAT3 antibody (*cat#: 9139*), anti-p-STAT3 (Y705) antibody (*cat#: 9145*), anti-cleaved caspase-3 antibody (*cat#: 9664*) were purchased from Cell Signaling Technology Inc., Danvers, MA, USA. All primary antibodies were diluted at 1:500.

### Co-IP analysis

The cells were lysed using IP lysis buffer (150 mM NaCl, 25 mM Tris-HCl at pH 7.4, 1 mM EDTA, and 1% NP-40) for 30 min on ice. The supernatant was harvested by centrifuge at 14,000 × g for 10 min at 4 °C. 20 µl of A/G sepharose beads and 2 µg of rabbit IgG control antibody was added to a clean EP tube containing 2 mg protein. The sample was rotated for 1 h at 4 °C, followed by centrifuge at 1000 × g for 1 min at 4 °C to pellet the IgG bound-A/G sepharose beads. Then the bead pellet was discarded, and the supernatant was transferred to a fresh EP tube containing either 5 µg rabbit IgG control antibody *(cat# SP034*, *Solarbio Life Science*, *Beijing*, *China*) or 5 µg anti-PDK1 antibody at a dilution 1:40 (*cat# 3820*, *Cell Signaling Technology Inc*., *Danvers*, *MA*, *USA*) plus A/G sepharose beads (40 µl) or A/G sepharose beads only. The IP lysis buffer was added to the mixture until the final volume was 500 µl. Then the sample was rotated overnight at 4 °C. To collect the immunoprecipitated protein, the sample was centrifuged at 1000 g for 1 min at 4 °C to pellet the antibody-bound A/G sepharose beads, and the supernatant was aspirated. The beads were re-suspended in 1 ml of IP lysis buffer and centrifuged at 1000 g for 1 min at 4 °C, and the supernatant was discarded, and this procedure was repeated for 3 times. Finally, the beads was resuspended in 6 × laemmli buffer, boiled for 5 min at 100 °C, which was used to perform the SDS-PAGE western blot to detect the STAT3 protein.

### Lentiviral package

PDK1 shRNA plasmids were extracted with an endo-free max kit (*Omega*, *USA*). The shuttle plasmids were mixed with the L1, L2, and VSVG package plasmids, which were transferred to 293FT. Viral supernatant was collected after 48 h, filtered through a 0.45 µm filter, and centrifuged at 75,000 g for 90 min at 4 °C. Next, the supernatant was discarded, and the pellet (lentiviral particles) was resuspended with PBS buffer. The stable cell line was screened and selected with puromycin for 2 weeks.

### Xenograft animal model

All nude mice and BALB/C mice were housed and cared for according to the guidelines established by the Animal Care Committee of Sun Yat-Sen University, and the experimental protocol was approved by Medical School of Sun Yat-Sen University. Female nude mice (4–5 weeks old) were housed in a specific pathogen-free barrier system, and female BALB/C mice (7–8 weeks old) were maintained in environmentally controlled conditions. 8 × 10^6^ HCT116 cells were subcutaneously injected into the right flank of nude mice, and liver metastasis models (nude mice and BALB/C mice) were constructed through spleen injection of HCT116 cells (5 × 10^6^ or 2.5 × 10^6^). SH-4-54 (an inhibitor of total STAT3, 0.2 mg/d/mouse) or CPT (2 mg/ml, 0.2 mg/d/mouse) were injected (i.p.) every other day (day 1, 3, 5, 7) for a total of four times. 50 µl DCA (0.5 mol/L) was administered (i.p.) every day (day 1–8) for a total of eight times. The volume of subcutaneous tumors was measured for 2 weeks and calculated as Volume (mm^3^) = (π × Length × Width × High)/6. The liver metastatic area was calculated by ImageJ software.

### Statistical analysis

The continuous variables were analyzed using Student’s t-test. The survival curves for patients were analyzed by the STATA 15.1, and the growth curve for the nude mice model was compared using two-way ANOVA. All statistical analyses were performed using SPSS 11.0 software, and *P* < 0.05 was considered to be statistically significant.

## Supplementary information

Supplementary material

## Data Availability

All data generated or analyzed during this study are included in this published article (and its Supplementary Information Files).
