# Supplementary material for: The double inhibition of PDK1 and STAT3-Y705 prevents liver metastasis in colorectal cancer
Source: Sci Rep. 2019 Sep 10;9:12973. doi: 10.1038/s41598-019-49480-8 (PMC6736869; doi:10.1038/s41598-019-49480-8)
Supplement: Supplementary file 1 — Supplementary material [file 41598_2019_49480_MOESM1_ESM.pdf]

The double inhibition of PDK1 and STAT3-Y705 prevents liver metastasis in colorectal cancer

Wenjuan Qin, Yun Tian, Jing Zhang, Wenjian Liu, Qiming Zhou, Sheng Hu, Fei Yang, Li Lu, Haijie Lu, Shuzhong Cui, Lu Wen, Shaozhong Wei

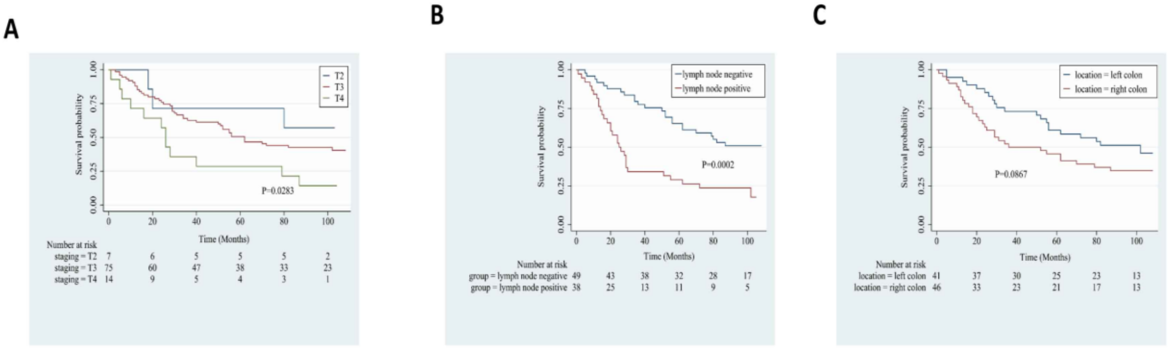

Fig. S1 The comparison of overall survival in patients (N = 100) with CRC according to different pathological characteristics. T2 and T3 stage (A), no lymph node metastasis (B) and left-sided tumor (C) showed a favorable prognosis than T4 stage, lymph node metastasis and right-sided tumors, respectively (All *P* values were less than 0.05).

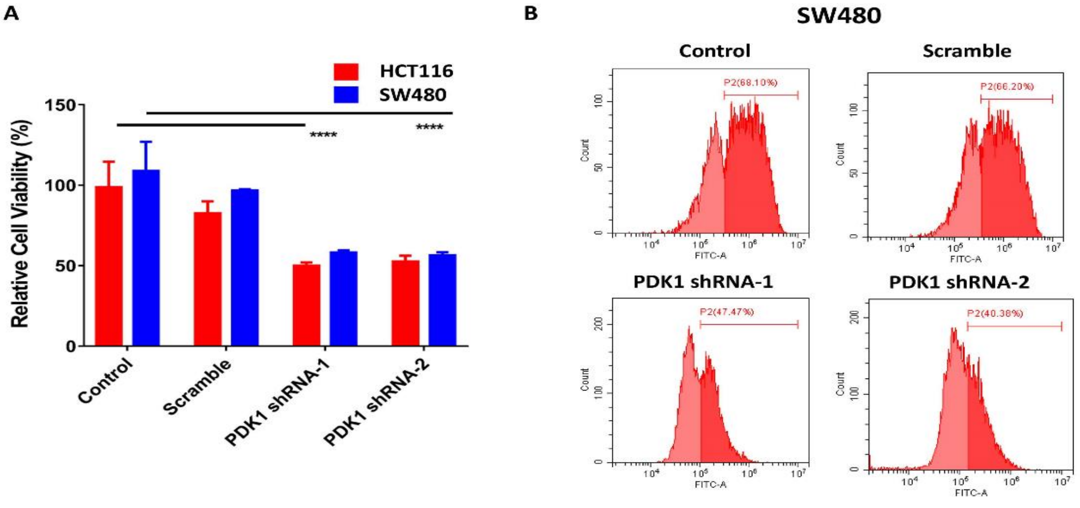

Fig. S2 Silencing PDK1 decreased the CRC cell viability and proliferation. A: CCK-8 cytotoxicity assay showed both HCT116 and SW480 cells had a significantly reduced cell viability when PDK1 was silenced. Data expressed as mean ± S.D., **** represents $P < 0.0001$. B: A representative EdU incorporation assay demonstrated knockdown of PDK1 significantly suppressed the proliferation of SW480 cells.

Table S1 The clinical characteristics in patients with low PDK1 expression and high PDK1 expression.

**Factors**	**Low PDK1 expression (n=37)**	**High PDK1 expression (n=42)**	***P***
Gender			0.7618
Male	19	23	
Female	18	19	
Age (years)			0.1722
<60	12	8	
≥60	25	34	
Tumor Size			0.360
T2	2	3	
T3	30	28	
T4	5	11	
Location			0.3553
Left colon	17	15	
Right colon	20	27	
Lymph node metastasis			0.8242
Negative	22	26	
Positive	15	16	
Liver metastasis			0.9999
Yes	1	1	
No	36	41	

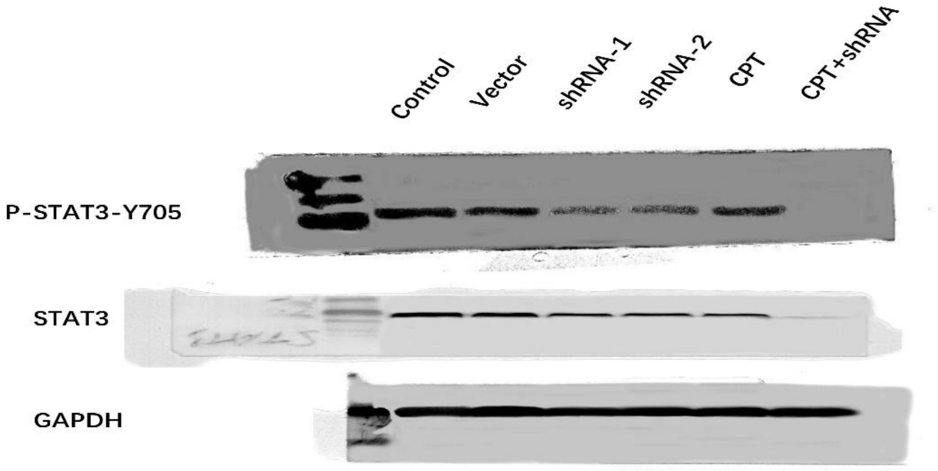

Fig. S3 The original imaging of the cropped western blotting in Fig. 2E.

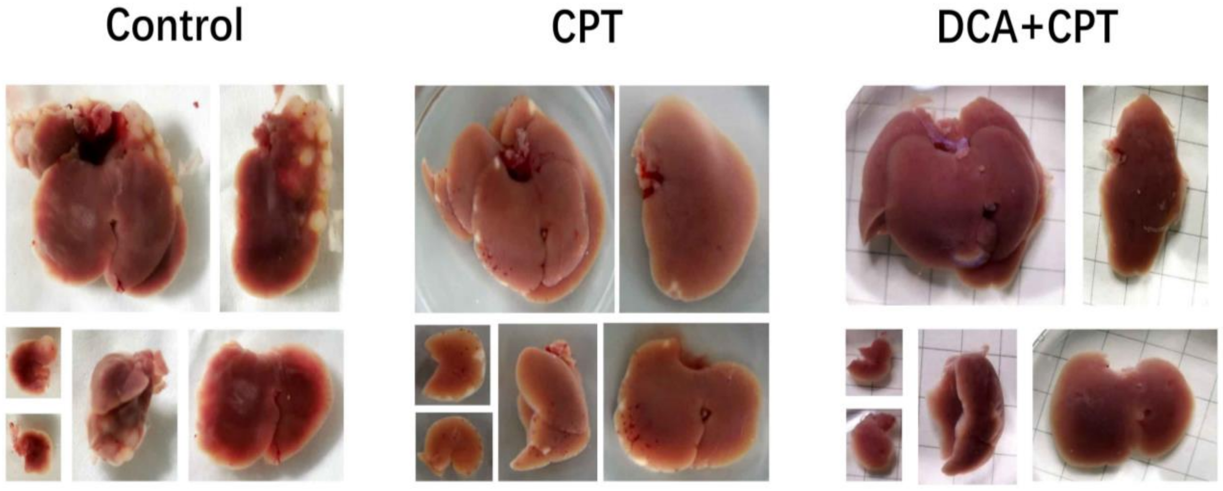

Fig. S4 CPT with or without DCA significantly reduces liver metastasis of CRC. 2.5×10^6 HCT116 cells were injected into the spleens of immune-competent BALB/C mice under isoflurane anesthesia on day 1. CPT (0.2 mg/d/mouse) was administered (i.p.) every other day (day 1, 3, 5, 7) for a total of four times. 50 μl DCA (0.5 mol/L) was administered (i.p.) every day (day 1-8) for a total of eight times. PBS was administered in the control mice. On day 9, these mice were sacrificed.

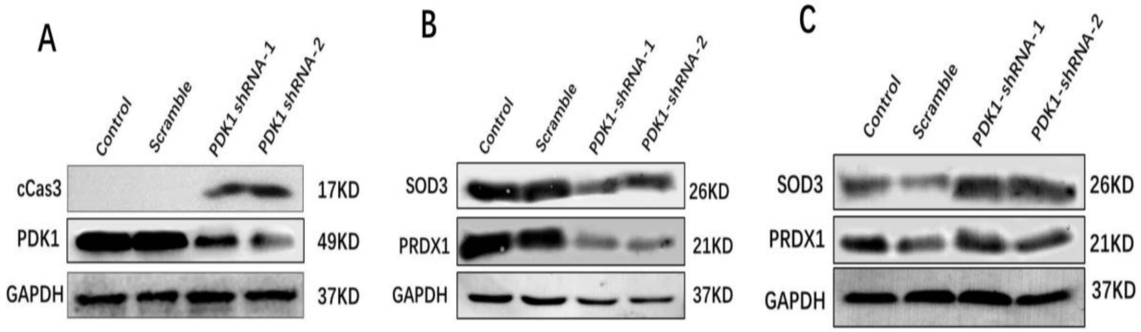

Fig. S5 Knockdown of PDK1 upregulates the expression of cleaved caspase-3 and affects the expression of antioxidative genes by western blot. A: PDK1 knockdown significantly inhibited the expression of PDK1 and promoted the expression of cleaved caspase-3 in HCT116 cells. Two gels were loaded, and blots from different proteins were cropped and grouped into one image with white area separated in between different proteins. The exposure time was 40 s, 25 s, 10 s for cleaved caspase-3, PDK1 and GAPDH, respectively. B: In normal culture condition, PDK1 knockdown downregulated the expression of SOD3 and PRDX1. Three gels were loaded, and blots from different proteins were cropped and grouped into one image with white area separated in between different proteins. The exposure time was 45 s, 30 s, 10 s for SOD3, PRDX1 and GAPDH, respectively. C: In anoikis culture condition, PDK1 knockdown increased the expression of SOD3 and PRDX1. Three gels were loaded, and blots from different proteins were cropped and grouped into one image with white area separated in between different proteins. The exposure time was 45 s, 30 s, 10 s for SOD3, PRDX1 and GAPDH, respectively.
